# Insights into the secondary and tertiary structure of the Bovine Viral Diarrhea Virus Internal Ribosome Entry Site

**DOI:** 10.1080/15476286.2022.2058818

**Published:** 2022-04-05

**Authors:** Devadatta Gosavi, Iwona Wower, Irene K. Beckmann, Ivo L. Hofacker, Jacek Wower, Michael T. Wolfinger, Joanna Sztuba-Solinska

**Affiliations:** a Department of Biological Sciences, Auburn University, 120 W. Samford Ave, Rouse Life Sciences Building, Auburn, AL, United States; b Department of Animal and Dairy Sciences, Auburn University, Auburn, AL, United States; c Department of Theoretical Chemistry, University of Vienna, Vienna, Austria; d Research Group Bioinformatics and Computational Biology, Faculty of Computer Science, University of Vienna, Vienna, Austria; e Institute of Bioorganic Chemistry, Polish Academy of Sciences, Poznan, Poland

**Keywords:** Internal ribosome entry site (IRES), bovine viral diarrhoea virus (BVDV), selective 2′ - hydroxyl acylation analysed by primer extension and mutational profiling (SHAPE-MaP), H-type pseudoknot, covariance analysis

## Abstract

The internal ribosome entry site (IRES) RNA of bovine viral diarrhoea virus (BVDV), an economically significant *Pestivirus*, is required for the cap-independent translation of viral genomic RNA. Thus, it is essential for viral replication and pathogenesis. We applied a combination of high-throughput biochemical RNA structure probing (SHAPE-MaP) and *in silico* modelling approaches to gain insight into the secondary and tertiary structures of BVDV IRES RNA. Our study demonstrated that BVDV IRES RNA in solution forms a modular architecture composed of three distinct structural domains (I–III). Two regions within domain III are represented in tertiary interactions to form an H-type pseudoknot. Computational modelling of the pseudoknot motif provided a fine-grained picture of the tertiary structure and local arrangement of helices in the BVDV IRES. Furthermore, comparative genomics and consensus structure predictions revealed that the pseudoknot is evolutionarily conserved among many *Pestivirus* species. These studies provide detailed insight into the structural arrangement of BVDV IRES RNA H-type pseudoknot and encompassing motifs that likely contribute to the optimal functionality of viral cap-independent translation element.

## Introduction

Bovine viral diarrhoea virus (BVDV) is a member of the genus *Pestivirus*, family Flaviviridae, that includes the causative agents of economically significant diseases of cattle, pigs, and sheep. The BVDV infections in cattle lead to decreased fertility and milk production, slow foetal growth, diarrhoea, respiratory symptoms, reproductive and immunological dysfunction [1]. According to the ICTV virus taxonomy profile [2], two main genotypes of BVDV, namely type 1 (*Pestivirus* A) of low-virulence and type 2 (*Pestivirus* B) of high-virulence, have been recognized and are estimated to cause considerable economic loss [3]. Besides the two BVDV genotypes, several genetic isolates have been distinguished within BVDV type 1 [4,5].

The BVDV genome is a positive-sense single-stranded RNA (12.5 kb) that codes for one open reading frame (ORF). The coding region is preceded by the distinctly structured 5′ untranslated region (5′ UTR), which folds into the Internal Ribosomal Entry Site (IRES) [6]. By definition, IRES is the RNA domain that recruits ribosomes to the internal region of mRNAs to initiate translation through a cap-independent pathway. IRES domains can be found in genomic RNAs of many pathogenic viruses. In Hepatitis C virus (HCV) genomic RNA, it mediates the initiation of translation by recruiting the subset of canonical translation initiation factors (eIF2, eIF3), methionine tRNA (Met-tRNAi), and by orienting the 40S ribosomal subunit at the translational initiation codon [7,8]. Cricket paralysis virus (CrPV) IRES RNA directs ribosomes without the requirement of additional translational factors [9], while IRES RNAs of poliovirus (PV) [10] and foot and mouth disease virus (FMDV) [11] rely on binding of eIFs, Met-tRNAi, and additional proteins referred to as the IRES trans-acting factors (ITAFs) [5,12–14]

According to previous structural studies, the viral IRES RNAs fold into intricate secondary and tertiary arrangements that provide a structural scaffold for cellular initiation factors and trigger conformational changes in the 40S ribosomal that drive translation initiation by an active mechanism [15–18]. For example, the HCV IRES consists of four structurally defined domains (I–IV), with domain II inducing the open conformation of the 40S subunit and III and IV forming a functionally essential pseudoknot [19]. Pseudoknots are formed upon base-pairing of a single-stranded region of RNA in the loop of a hairpin or a bulge to a stretch of complementary nucleotides elsewhere in the RNA chain. The flexibility and transient formation of pseudoknots might be essential for recognition and favourable interaction with translating ribosomes, or the translation factors associated with the ribosomes [20]. Thus, the folding strategy can generate a vast number of three-dimensional folds, which exhibit a diverse range of highly specific functions [21]. CrPV IRES also displays a multi-domain composition with the residues of one domain supporting a pseudoknot. In the case of BVDV IRES (SD-1 isolate, genotype 1), thermodynamic and phylogenetic studies suggested that its translational functionality is also controlled by the formation of a pseudoknot [21,22]. Burks et al., using comparative structural studies, predicted that the BVDV IRES pseudoknot involves interaction between the loops of hairpin 3 and 4 within domain III [5,22,23]. Toeprinting *in vitro* analysis involving BVDV IRES, 40S rRNA, and eIFs suggested that the ribosome makes multiple contacts with the BVDV IRES. Similar observations have been made for HCV IRES RNA [24–27], outlining particularly interesting contacts existing between 18S rRNA and the pseudoknot formed within IRES domain III [18,23]. Additionally, the presence of an RNA pseudoknot in BVDV IRES RNA was supported by compensatory mutations, which restored translation [22].

In this study, we investigated the secondary structure of BVDV IRES RNA using selective 2′-hydroxyl acylation analysed by primer extension and mutational profiling (SHAPE-MaP) [28,29]. Our analysis, which has been performed on two *in vitro* synthesized constructs that contain all essential elements supporting the cap-independent translation, revealed that BVDV IRES RNA folds into three distinct structural domains (I–III). Two single-stranded regions of domain III were shown to support the formation of an H-type pseudoknot, thus corroborating earlier studies that predicted the existence of a pseudoknot in BVDV IRES RNA and in related pestiviruses [30,31]. To obtain a better picture of pseudoknot formation, we performed RNA 3D simulations with ernwin [32] and SimRNA [33] on a trimmed construct of the BVDV-1 strain NADL IRES. Tertiary structure simulations confirmed that an H-type pseudoknot can be formed under the condition that two adjacent helices are oriented in a well-defined range of angles against each other. On a broader scale, we performed a comparative genomics screen of the 5′ UTR in the genus *Pestivirus*, which confirmed pervasive evolutionary conservation of the IRES region, and provided evidence for the presence of a pseudoknot in all studied viruses.

## Results

### The secondary structure of BVDV IRES RNA

SHAPE-MaP is a high-throughput biochemical probing technique that can be used to characterize RNA secondary structure at a single-nucleotide resolution (Fig. S1). In SHAPE-MaP, unpaired nucleotides (nts) are acylated more readily at the 2′-hydroxyl ribose moieties by an electrophilic reagent, e.g. 1-methyl-7-nitroisatoic anhydride (1M7) [34] than those that are paired, and the reactivity biases are employed to address RNA secondary structure [35]. The modified RNA is directed to reverse transcription reaction in the presence of Mn^2+^ ions that induce reverse transcriptase to read through the bulky 2′-O-adducts and insert mutations [36]. The resulting cDNA products carrying the mutations are directed to stepwise amplification to generate DNA libraries, which are directed to next-generation sequencing.

We performed structural probing of BVDV IRES RNA on two *in vitro* synthesized transcripts; the short transcript (560 nts) included the BVDV IRES RNA sequence only; the long transcript (1296 nts) additionally contained the EGFP cassette (nts 561–1296), and the MS2 hairpin (nts 523–543) at its 3′ end. The IRES RNA constructs used in this study were derived from BVDV-1 strain NADL genomic RNA and contain RNA sequences that were outlined as necessary for supporting the expression of EGFP protein by the cap-independent translation [37,38]. BVDV-1 strain NADL is a cytopathic strain, originally isolated from an animal infected with fatal mucosal disease [39] (Fig. S2). We confirmed the homogeneous conformation of both folded RNA constructs by native gel electrophoresis prior to structural probing (Fig. S3). The comparison of SHAPE reactivity profiles for short and long IRES RNA constructs, performed by custom R scripts, showed a positive correlation established by an average Pearson correlation coefficient of 0.8398 (*p*-value 0.022) (Fig. S4). Since we obtained a more comprehensive mapping output for the long transcript, we used these data for modelling the BVDV IRES RNA.

**Figure 1.**
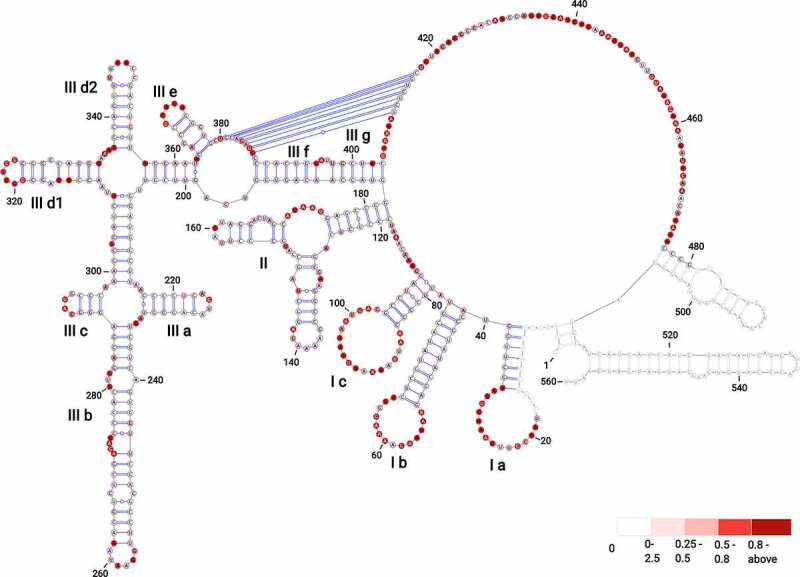
The secondary structure model of BVDV IRES RNA derived from SHAPE-MaP probing. The three main domains are labelled (I–III) with outlined individual RNA motifs: SL Ia, SL Ib, SL Ic, SL II, SL IIIa, SL IIIb, SL IIIc, SL IIId1, SL IIId2, SL IIIe, SL IIIf, SL IIIg. The H-type pseudoknot has been manually inserted within the single-stranded residues at positions 381–387 and 410–416 of domain III. The residues lacking reactivity values correspond to the sites of primer annealing (light grey). The secondary structure is colour-coded according to the SHAPE reactivity values represented by low (< 0.25), medium (0.25–0.80), high reactivity (0.8–2), and hyperreactivity (>2).

Our analysis revealed that the BVDV IRES can be divided into three domains labelled I–III, with individual stem-loops (SL) within each domain being assigned letter codes (Figure. 1). Domain I contain SL Ia, which includes an apical loop with an equal number of purines and pyrimidines, SL Ib, which has a purine-rich apical loop, and SL Ic, which has an adenine-rich apical loop. Domain II forms a Y-shaped hairpin that includes two SLs interconnected by a three-way junction. The 5′ SL folds into two short stems interrupted by an asymmetrical internal loop with three nts at the 5′ side and mispaired adenine at the 3′ side. The apical loop includes predominantly adenines, which are largely not reactive to SHAPE reagent, likely due to extensive base stacking interactions characteristic for A-rich sequences [40]. The 3′ SL contains a 3′ four nts bulge and a UNRN tetraloop (UAGU), where N stands for any nucleotide and R is a purine base. These types of tetra-loops are frequently present in apical loops involved in the U-turn formation, where the backbone changes direction immediately following the initiating U, and the Watson–Crick faces of the bases immediately 3′ of the U are exposed to the solvent [41,42]. Domain III includes the main stem that is formed by SL IIIf and IIIg, interrupted by a 2 nt bulge, followed by a three-way junction that connects SL IIIe and the apical IRES portion. Successively, a four-way junction connecting SL IIId1 and IIId2 is present, followed by a four-way junction connecting SL IIIa, b, and c. The stem of SL IIIa includes an AGUA tetra-loop with less-reactive UA residues [41]. The SL IIIb forms the longest hairpin of this region, closed with a purine-rich apical loop. It includes an asymmetrical internal loop at its 3′ side, a downstream 3 nts bulge, and a cytosine mismatch. The SL IIIc includes three canonical base pairs and a CAUG tetra-loop belonging to the YNMG tetra-loop family, where Y stands for U/C, N, and M is for A/C/U. Previous NMR, circular dichroism, and functional group substitution studies in 16S rRNA indicated the unusual thermodynamic stability of YNMG tetra-loop and showed its involvement in tertiary interactions [43]. SL IIId1 is closed with an apical penta-loop that consists of highly reactive G residues (median SHAPE reactivity of 1.020). This type of G-rich apical loop was found in the 5′ UTR SL2 and SL3 of the HIV-1 genome involved in binding the nucleocapsid protein [44]. The SL IIId2 involves a stem with two GU wobble base pairs and a 6 nt apical pyrimidine-rich loop. The SL IIIe is closed with a UGAUA penta-loop.

The residues immediately downstream of SL IIIe and the residues near the AUG codon (nts 429–431) were proposed previously to be involved in a H-type pseudoknot [21,22]. In the structure predicted from SuperFold, however, part of these residues was involved in the formation of two hairpins held by two reactive GC pairs (G381, reactivity value of 0.219 pairing with C388, reactivity value of 0.408; G387, reactivity value of 0.714 pairing with C382, reactivity value of 0.175 for hairpin 1; and G415, reactivity value of 0.121 pairing with C426, reactivity value of 0.57; G425, reactivity value of 0.346 pairing with C416, having no reactivity value for hairpin 2). Considering that cytosines are known to be the least reactive towards SHAPE electrophilic reagents and are generally involved in single-stranded regions [45,46], we resolved these hairpins. Subsequently, the pseudoknot in domain III has been manually inserted within the single-stranded regions at positions 381–387 (5′-GCAGAGG-3′, median SHAPE reactivity of 0.617) and 410–416 (5′-UCUCUGC-3′, median SHAPE reactivity of 0.115).

The single-stranded region immediately downstream of the pseudoknot harbours the AUG codon. We noted that numerous residues within BVDV IRES RNA display hyperreactivity towards SHAPE reagents (Table S1, Fig. S4). These are found within the loops (nts 26, 22, 54, 58, 60, 91, 99), bulges (nts 271, 273), single-stranded regions (nts 116, 438, 444, 455, 461, 465, 469), and near the pseudoknot (nts 409, 417) (Fig. S5). These residues were previously reported as accommodating a C2-endo ribose conformation [47]. This conformation of ribose shows slow dynamics and favours intra-residue H bonding between the 2′-hydroxyl and the 3′-oxygen, enhancing the reactivity of the 2′-hydroxyl towards SHAPE reagent [48].

**Figure 2.**
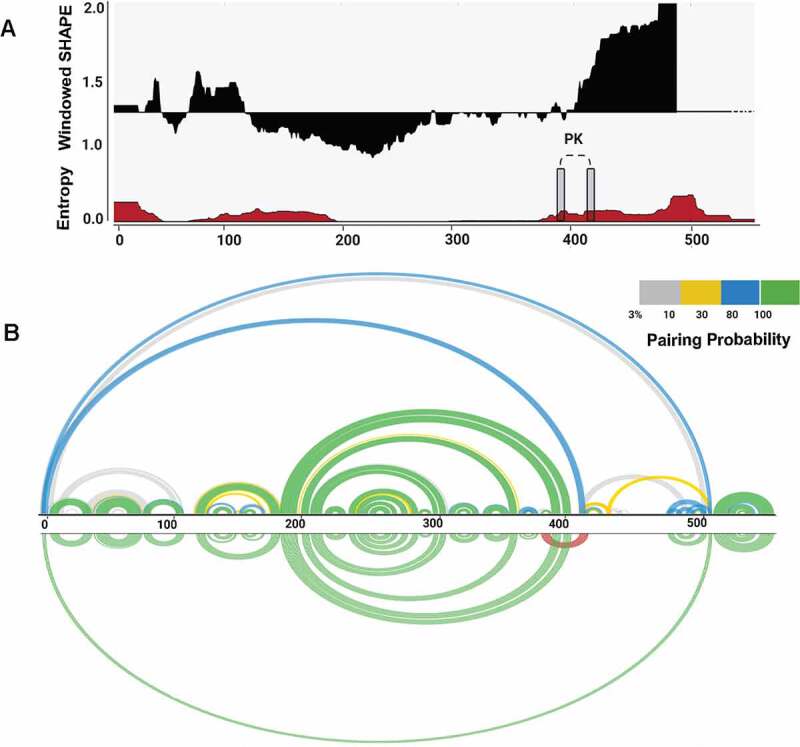
Structural features of BVDV IRES RNA. (A) Windowed SHAPE reactivities (black) and Shannon entropies (brown) are indicated. The X-axis represents the nucleotide position, and Y-axis corresponds to windowed SHAPE reactivities (top) and entropy values (bottom). Single-stranded regions involved in pseudoknot formation are indicated with grey rectangles. (B) Arc plots represent the base-pairing probabilities for BVDV IRES RNA. The top arc plot is colour-coded according to the pairing probability scale. The bottom arc plot includes the predicted H-type pseudoknot (red).

We used SuperFold [29] to compute a SHAPE-MaP assisted minimum free energy structure, and base pair probabilities of the BVDV IRES RNA by partition function folding. SHAPE reactivities are incorporated into the folding recursions as pseudo energies, following the approach proposed by Deigan et al. [49]. The obtained model was visualized as a colour-coded arch plot (Figure. 2). Well-defined pairings outlined by high pairing probability between 0.8 and 1.0 (green arcs) are visible in regions folding into SL Ia – c and SL IIIa – e. Regions with low pairing probabilities <0.3 (yellow and grey arcs) are scarce and dispersed among residues that, per SHAPE modelling, are predicted to be single-stranded. Overall, the arc model displayed strong agreement between nucleotide-resolution experimental SHAPE reactivity data and modelled base-pairing. We also assessed how accurately the secondary structure motifs are defined by their sequence and the probing data by calculating the Shannon entropies at nucleotide resolution [36,50]. We noted that the region corresponding to the Domain III (nts 200–380) displays low SHAPE reactivity values, low Shannon entropy values, and high pairing probability, which likely reflects its structural and functional significance. On the other hand, the regions that display low (nts 130–200) and high (nts 420–490) SHAPE reactivity values and high Shannon entropy correspond to Domain II and single-stranded residues of Domain III that represent the formation of a pseudoknot (nts 381–387 and 410–416). These parameters were shown previously to be characteristic of flexible regions that can fold into interconverting RNA structures [51,52].

### Evolutionary conservation of pseudoknot in the genus *Pestivirus*

**Figure 3.**
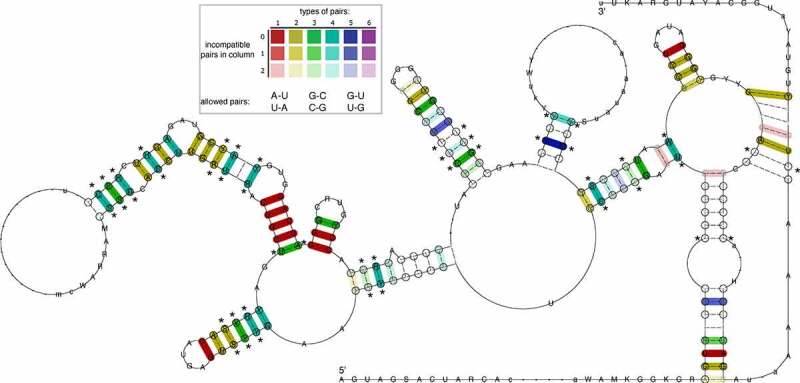
The consensus of IRES Domain III secondary structure prediction generated for 13 *Pestivirus* genomes highlights rich covariation patterns by the different colouring of base pairs, following the RNAalifold colour scheme. Red indicates nucleotide-level sequence conservation, while other colours highlight increasing covariation levels by structure-conserving nucleotide substitutes, as depicted in the insert. The model includes nucleotides with a frequency greater than the average in the underlying alignment in IUPAC notation. Dashes along the consensus sequence indicate positions where the majority of sequences had gaps. Circled nucleotides highlight compensatory mutations, i.e. cases where both nucleotides of a base pair are mutated, such as UA -> GC. Statistical significantly covarying base pairs are marked with an asterisk.

To obtain deeper insight into the evolutionary conservation of the BVDV IRES region, and in particular conservation of the pseudoknot, we performed a comparative genomics screen within the genus *Pestivirus*. To this end, we selected 13 representative viral genomes, comprising the ICTV-approved *Pestivirus* species A-K, as well as Linda virus and Norway Rat *Pestivirus*, and searched for hits of the Rfam *Pestiviru*s IRES (RF00209) covariance model. Only high-quality (high bit score) hits for each input sequence were considered. Multiple sequence alignment (Fig. S6), and consensus structure prediction of these structurally homologous elements revealed rich covariation patterns throughout the entire IRES region (Figure. 3). A test for statistically significantly covarying base pairs with R-scape reported 23 hits, one of them in the pseudoknot.

### Comparison of probing-assisted structure model of BVDV IRES RNA with previously proposed models

We performed a comparative analysis of our probing-assisted BVDV IRES RNA structure model and previously proposed models, which revealed both commonalities and differences (Fig. S7). In the BVDV IRES model that was proposed by Deng and Brock, a largely equivalent architecture of domain III computed by early versions of modern RNA structure prediction algorithms highlights its functional importance [53]. Likewise, prediction of pseudoknot formation in domain III on a shorter construct by Le et al., is in agreement with our experimental data [30]. Contrary, domain II exhibits a lower degree of similarity. While our prediction is in favour of a Y-shaped three-way junction element (Figure. 1), earlier studies reported an extended stem-loop structure that is interspersed with bulges. We used homologous sequences from the 5′ UTR of ICTV-approved *Pestivirus* species A-K to construct a consensus structure of domain II. Interestingly, an extended stem-loop structure is supported by multiple covariations in the consensus model (Fig. S8). Partition function folding and re-evaluation of the corresponding element in our model revealed that domain II encompasses two entities, i.e. a structurally constrained closing stem, as well as a conformationally flexible apical portion that can fold into either an extended stem-loop or a Y-shaped structure (Fig. S9). As these conformations co-exist within a narrow energy range of 1 kcal/mol, we consider the apical portion of domain II is multi-stable. Taken together, earlier models of the BVDV-1 NADL IRES RNA, in particular domain III stay, to a large extent, in agreement with our SHAPE-MaP assisted structural analysis (Supplemental Data: https://github.com/jzs0165/BVDV-Files)

### The modelling of BVDV IRES RNA 3D structure

SHAPE-assisted secondary structure prediction reported the existence of an H-type pseudoknot near the basal stem of Domain III. By analysing the predicted secondary structure, one might expect a co-linear arrangement of SL IIIf and IIIg, resulting in coaxial stacking of the two helices. However, under such a scenario, spatial separation of the complementary pseudoknot interaction regions induced by the putatively coaxially stacked helical stems SL IIIf and SL IIIg might be too large to form the pseudoknot. We were intrigued by the question of whether this pseudoknot interaction is sterically feasible in 3D.

To this end, we performed coarse-grained RNA 3D simulations with a purposefully trimmed construct that encompasses relevant parts of Domain III that are involved in pseudoknot formation. Specifically, 5′ and 3′ portions of the original construct were truncated after positions 179 and 425, respectively. Likewise, the four-way junction that contains SL IIId1, SL IIId2, and the substructure comprising SL IIIa, b, and c, were retained as an apical loop element with a connected backbone. This construct, denoted BVDVsegment_180_425 (Fig. S10), features the same structural traits as the original full-length construct. In particular, pKiss single sequence folding predictions suggest the formation of an H-type pseudoknot as expected. Likewise, this truncated construct represents a reasonably small system that allows for detailed tertiary structure analysis *in silico*.

We used ernwin to test whether the proposed secondary structure can be embedded in 3D. Operating on secondary structure elements, ernwin employs a coarse-grained sampling approach and distinguishes between stems, bulges, and various loop types, each represented by cylinders that fit the helix axis. 3D structures are built from fragments of already known RNAs, and tertiary structures are guaranteed to always match the input secondary structure. Building on this concept, we constructed initial 3D models of BVDVsegment_180_425, thereby confirming that the proposed H-type pseudoknot is sterically feasible.

**Figure 4.**
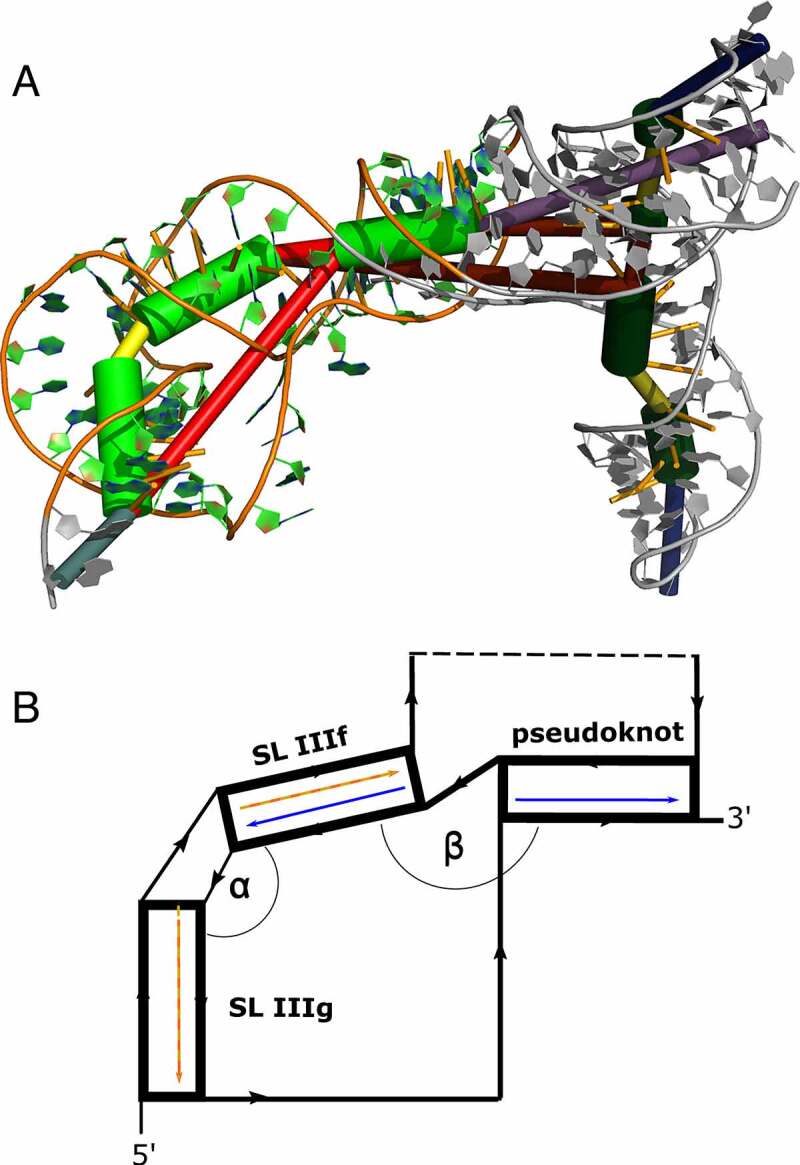
(A) Rendering of a low-energy tertiary structure snapshot of the truncated construct BVDVsegment_180_425 overlaid with coarse-grained 3D elements. Green cylinders highlight helical regions, with the rightmost representing H-type pseudoknot, and the middle and leftmost green cylinders portraying stem-loops IIIf and IIIg, respectively. The bulge between SL IIIf and IIIg is depicted in yellow, and the multiloop enclosed by the formation of the pseudoknot is plotted as a long red connection. The short red connection between the pseudoknot helix and SL IIIf represents a single unpaired C between these helices. Other structural elements are greyed out. The quasi-colinear arrangement of the pseudoknot and SL IIIf is noticeable, while the bulge induces a marked twist between SL IIIf and IIIg. (B) Schematic representation of the three helices encompassing the pseudoknot, SL IIIf, and IIIg, sketching their approximate spatial arrangement. Angles between adjacent helical segments are defined by stem vectors, i.e. angle α between SL IIIf and SL IIIg (orange and red vectors), and β between SL IIIf and the pseudoknot helix (blue vectors). The directionality of the backbone is indicated by black arrows. Regions of BVDVsegment_180_425, which are not directly involved in the spatial arrangement of these helices, are depicted as a dashed segment along the backbone.

**Figure 5.**
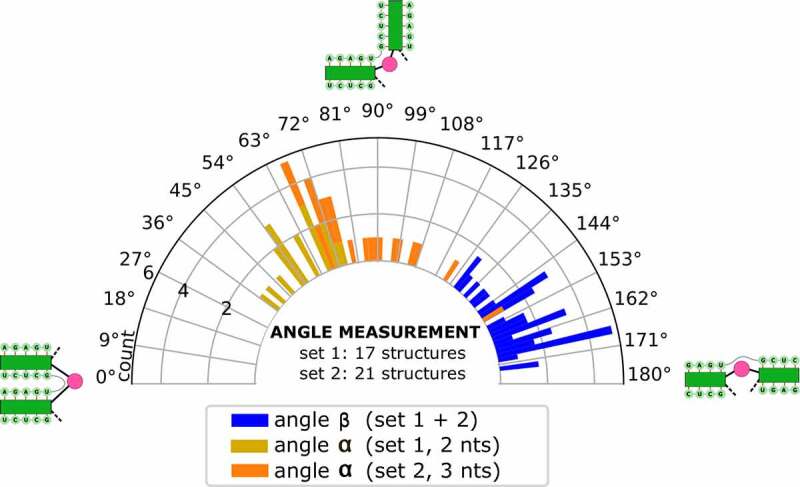
Distribution of angles α and β in the low-energy 3D structure of BVDVsegment_180_425. The relative orientation of helices was assessed by a helix-centred representation of RNA 3D structure. As stretched by the relative geometry of green helical segments, angles close to 0° account for parallel stems, whereas angles close to 180° indicate collinear arrangement and can be indicative of coaxial stacking. Analysis of candidate structures from SimRNA simulations of BVDVsegment_180_425 shows that angles between helices are distributed in well-defined intervals. Set 1 and set 2 comprise low-energy structures predicted by SimRNA, whose helices SL IIIf and IIIg are separated by a bulge of 2 and 3 nts, respectively. For set 1, α lies between 37 and 75 degrees, while for set 2, values are distributed between 66 and 149 degrees. This is expected, as a larger bulge allows for more conformational flexibility of the two adjacent helices. Contrary, β falls in a range between 130 and 174 degrees for set 1 and 129 and 173 degrees for set 2. Values for both sets were combined and depicted in blue.

In parallel, we set out to predict the tertiary structure of BVDVsegment_180_425 with SimRNA. We performed massively parallel replica-exchange Monte Carlo simulations to sample the low-energy portion of the underlying energy landscape, using secondary structure restraints that ensure the formation of all helices predicted for BVDVsegment_180_425, including the pseudoknot. A snapshot of a low-energy conformation is shown in (Figure. 4). Clustering of predicted low-energy structures allowed us to characterize two sets of highly compact target structures, 38 in total, that differ in the size of the bulge between helical segments IIIf and IIIg, i.e. 2 nt in set 1 (17 candidate structures) and 3 nt in set 2 (21 candidate structures). The sets of candidate structures were then subjected to follow-up analysis with forgi [54], focusing on the angles between the pseudoknot helix and SL IIIf (angle β) and SL IIIf and SL IIIg (angle α). As shown in (Figure. 5), both angles are distributed in well-defined intervals for all 38 low-energy structures. While α falls between 37 and 149 degrees, β is dispersed between 129 and 174 degrees. This suggests that all 3D candidate structures are well-defined and that there is nearly quasi-coaxial stacking between the pseudoknot helix and SL IIIf. Likewise, our data indicate that there is a marked twist between SL IIIf and IIIg. Notably, structures with a 2 nt bulge are less arched than those with a 3 nt bulge, which is in good agreement with their expected behaviour. An important finding of this analysis is that the pseudoknot can only be formed when SL IIIf and SL IIIg are oriented at an acute angle, facilitated by the central bulge.

## Discussion

RNA viruses are known to use exceptionally thrifty molecular strategies to optimize their replication cycle. They often contain functional RNA motifs within non-coding regions that control crucial steps of their infectivity cycle and structural switches that aid their regulation. The IRES RNA within the 5′ UTRs of BVDV is one of such complex structural domains known to govern the efficiency of cap-independent translation [23]. In this study, we applied RNA structural probing method and computational 3D modelling to gain insight into the secondary and tertiary structure of BVDV IRES RNA.

Overall, BVDV IRES RNA folds into three major structural domains (I–III), each containing well-defined motifs, i.e. hairpins and stems, interspersed among less structurally constrained regions, such as multi-way junctions and loops. This model stays, to a large extent, in agreement with the previously proposed secondary structure models for the 5′ and 3′ UTRs of BVDV strains, i.e. NADL, OSLOSS and SD-1 [5,30,53]. In particular, a good agreement for domain III folding has been found through the comparison of previous BVDV IRES structural models with our probing-assisted model. However, we have found an alternative folding of domain II into a Y-shaped hairpin instead of an extended hairpin, which can be justified by thermodynamic parameters, i.e. higher Shannon entropy and a small minimum free energy difference of 1 kcal/mol between both structures, which support the structural fluidity of domain II.

The key finding relates to the most structurally well-defined domain III (low SHAPE reactivities, low Shannon entropy values, high pairing probability), which includes two single-stranded regions and manually represented H-type pseudoknot. The 3D modelling of the BVDV IRES RNA provides further insight into the structural arrangements of individual motifs, predicting quasi-coaxial stacking between SL IIIf and the pseudoknot helix. These coaxial stackings can be critical for stabilizing the IRES pseudoknot and can be formed only if the angle between SL IIIg and SL IIIf is acute. Similar stabilization processes have been shown for T2 bacteriophage mRNA of gene 32 [55], turnip yellow mosaic virus (TYMV) [56], and simian retrovirus-1 (SRV-1) RNA pseudoknots [57]. The large junction connecting the individual RNA motifs of domains I–III likely provides a suitable movement and intrinsic flexibility to the single-stranded regions within domain III, which support the formation of a pseudoknot.

Previously, the formation of a pseudoknot in BVDV IRES RNA has been proposed based on thermodynamic predictions and Monte Carlo simulations [30,31]. The comparative phylogenetic analysis performed on BVDV IRES RNA sequences from genotypes 1 and 2 provided further evidence supporting the formation of a pseudoknot [5]. These studies showed that the pseudoknot engages between four and seven nucleotides, depending on the BVDV strain, with residues 5′-CAGA-3′ and 5′-UCUG-3′ being shared by all isolates. Here, we performed a computational screen of the IRES domain III in more than a dozen phylogenetically related viruses. Consensus structure prediction revealed rich covariation support in this evolutionarily conserved region of the *Pestivirus* IRES. Importantly, we could identify statistically significant covariation in 22 base pairs, including the pseudoknot, thus providing computational evidence for the crucial role of the pseudoknot in IRES functionality.

Site-directed mutagenesis analysis indicated that BVDV IRES RNA constructs with impaired base-pairing within these residues lost their translational activity, while compensatory mutations reversed that effect [22]. Furthermore, mutational and structural studies suggested the formation of structurally similar pseudoknots in the IRES of CSFV and HCV RNAs [58,59]. Additionally, it has been shown that the BVDV and HCV 5′ UTRs share a considerable extent of sequence and structure similarity [30,57,60]. The shared features include the formation of multiple SLs, which are globally organized into three domains surrounding the centrally positioned pseudoknot [61], with HCV IRES folding into additional domain IV, positioned externally [62]. Interestingly, in both HCV and BVDV IRES RNAs, domain III includes hairpins IIIa, IIId1, and IIIe, which apical loops, i.e. tetra-loop, G-rich loop, and penta-loop have highly conserved nucleotide sequence [19].

Although the relationship between the IRES pseudoknot and initiation codon in BVDV RNA has not been addressed, previous studies performed on HCV IRES RNA indicated that the formation of a pseudoknot leads to the correct positioning of mRNA open reading frame (ORF) in the 40S ribosomal binding cleft [63]. In HCV IRES, it was shown by SHAPE probing that upon binding of the 40S subunit P-site to the AUG codon, the residues at and beyond the start codon show a substantial increase in the flexibility of the translational initiation facilitation [64]. Similar structural probing experiments in the presence and absence of ribosomes can be performed to understand the effects of the translational machinery assembly on BVDV IRES RNA. Additionally, various therapeutic strategies involving the identification of small molecule inhibitors, antisense oligonucleotides, ribozymes, which target and disrupt the HCV IRES RNA and prevent the binding of translational factors, abolishing the viral replication, have also been developed [65]. Considering the similarity between HCV and BVDV IRES RNA, analogous strategies can be applied for interfering with BVDV replication.

## Materials and methods

### Cloning IRES from BVDV-NADL into pIW-IRES (eMS2hp)-EGFP expression vector

Oligonucleotide primers used in the study were purchased (IDT) and are listed in (Table S2). All DNA products were purified from agarose gels and eluted using the elution kit (Qiagen, 28,706). Quick Ligase (NEB, M2200L) was used in all ligation reactions. PCR reactions were carried using Q5 Hot Start Polymerase (NEB, M0493S).

### Cloning IRES under CMV promoter control

Inactivated BVDV-NADL virus was obtained from the laboratory of Paul Walz (Veterinary School, Auburn University). Viral RNA was extracted from 10^6^ viral particles following Ribozol extraction protocol (VWR Life Science, 97,064–950). Two hundred and fifty microlitres of virus suspension was mixed with three volumes of RiboZol and incubated for 5 min at room temperature. With an addition of 200 µl chloroform, the sample was shaken for 15 s and incubated for 15 min at room temperature. Phases were separated by centrifugation at 12,000 × *g* for 15 min in a cold microfuge. Six hundred microlitres aqueous phase was purified using Zymo Clean & Concentrator-25 (Zymo, R1018). Purified RNA (~1.2 µg) was used as a template for the synthesis of 461 bp DNA fragment coding for the 5′ UTR sequence and 25 codons of the first viral gene Npro. The oligonucleotide primers were designed using BVDV-NADL. cDNA was synthesized using 40 ng of viral DNA and 1 µM reverse primer IRES-NADL-rev in 20 µl reaction with SuperScript IV reverse transcriptase (Thermo Fisher Scientific, 18090010). The 461 bp DNA fragment was amplified using IRES-NADL forward and reverse primers and Q5 Hot Start polymerase (NEB, M0493S). The second round of PCR amplification extended the 461 bp fragment to include the KpnI restriction site at the 5′ end and the BamHI site at the 3′ end. Amplification with primer pair T7-IRES-for-3 and T7-NADL-rev yielded 509 bp DNA fragment, which was then digested with KpnI-HF (NEB, R3142S) and BamHI-HF (NEB, R3136S). The final 463 bp fragment was ligated with the pcDNA3.1(+)circ RNA Mini Vector (Addgene, 60,648) digested with KpnI-HF and BamHI-HF. Clones were selected and maintained in *E. coli* strain NEB5-alpha (NEB, C2987I). All constructs were verified by Sanger sequencing (Eurofins). New plasmid was designated pIW-IRES.

### Cloning gene for EGFP protein downstream from IRES

Plasmid pIRES-EGFP-puro (Addgene, 45567-DNA.cg) was digested with BstXI (NEB, R0113S) and BsrGI (NEB, R0575S), and 710 bp DNA fragment coding for EGFP protein was prepared for ligation. Plasmid pIW-IRES was digested with BamHI-HF (NEB, R3136S) and ApaI (NEB, R0114S). Insertion of the BstXI-BsrGI fragment with EGFP between BamHI and ApaI sites on the core plasmid was performed using the following adapters: A-5′EGFP-adapt-top and B-5′EGFP-adapt-bott for the 5′ end of the insert plus C-3′EGFP-top and D-3′EGFP-bott for the 3′ end of the insert. The 5′ end adapter pair added an EcoRI restriction site, while the 3′ end pair added an EcoRV site. Double digestion with EcoRI (NEB, R0101S) and EcoRV (NEB, R0195S) was used for the initial screening of clones. All putative correct clones were verified by sequencing. New plasmid was designated pIW-IRES-EGFP.

### Insertion of eMS2 hp at 3*′* end of IRES

The design of extended hairpin binding bacteriophage MS2 protein was described previously. Plasmid pIW-IRES-EGFP was linearized with BamHI-HF (the 3′ end of IRES) and BstXI (the 5′ end of EGFP). Gblock spanning the 3′ end of IRES, extended protein MS2-binding hairpin (eMS2hp), and the 5′ portion of EGFP were purchased (IDT) and amplified with primers R17L-for and R17L-rev gblock was digested with BamHI and BstXI and ligated to linearized pIW-IRES-EGFP. The sequence of plasmid pIW-IRES(eMS2hp)-EGFP was verified by sequencing.

### RNA isolation and purification

RNA was extracted with TRIzol reagent (Thermo Fisher Scientific, 15596018) followed by the RNA Clean and Concentrator kit (Zymo Research, 76020–604) and DNase treatment provided with the kit according to manufacturer’s protocols. The RNA was eluted in 15 µl RNAse free water and stored at −80°C.

### In vitro transcription

Plasmid pIW-IRES/eMS2hp-EGFP was linearized either with EcoRI for synthesis of IRES/eMS2hp, or with EcoRV for synthesis of IRES/eMS2-EGFP-mRNA. 100 µl transcription reaction consisted of 2.5 µg linearized plasmid, 200 mM HEPES-KOH, 7.5, 30 mM MgCl_2_, 40 mM dithiothreitol (DTT), 1 mM spermidine, 7.5 mM NTP, SUPERNase-In (Ambion) – 20 U/100 µl reaction, inorganic pyrophosphatase (NEB, M0361S) – 0.1 U/100 µl reaction and 100 µg/ml T7 RNA polymerase. The reactions were incubated for 2 h at 37°C with gentle rotation.

### BVDV IRES RNA SHAPE-MaP

The sequence corresponding to the BVDV IRES was divided into two overlapping zones (nts 1–283 and 217–504) that were reverse transcribed and amplified with zone-specific RT and PCR primers (Table S3). Five picomoles of BVDV IRES *in vitro* transcript (either short, 560 nts or long, 1296 nts) was heated at 95°C for 3 min and slowly cooled to 4°C (0.1°C/s). The *in vitro* transcripts were folded in a final volume of 36 µl in 1X folding buffer containing 50 mM Tris–HCl pH 8.0, 100 mM NaCl, 5 mM MgCl_2_, and incubated at 37°C for 15 min. The folded transcripts were divided into two reactions: positive and negative (18 µl each) with one sample treated with 2 µl of 100 mM 1-methyl-7-nitroisatoic anhydride (1M7) (DC Chemicals, 73043–80-8) for positive (+) reaction (final 1M7 concentration of 10 mM), and the second sample treated with 2 µl dimethyl sulphoxide (DMSO) for negative (-) reaction. Both tubes were incubated at 37°C for 5 min to facilitate RNA modification. Additionally, the denaturing control (DC) was prepared and included 5 pmol of transcript in 3 µl TE-like buffer (0.1 mM EDTA, 10 mM Tris-HCl, pH 7.5), 5 µl formamide, and 1 µl denaturing buffer (1 M HEPES, pH 8, 0.5 M EDTA), that were mixed and incubated at 95°C. To 9 µl of the denatured transcript, 1 µl of 100 mM 1M7 was added and incubated at 95°C for 1 min. All samples were purified using ethanol precipitation with 5 mg/ml glycogen (Thermo Fisher Scientific, AM9510). After modification, RNA was reverse transcribed at 42°C for 3 h using zone-specific RT primers in the SHAPE-MaP buffer, including 50 mM Tris pH 8, 75 mM KCl, 10 mM dithiothreitol (DTT), 6 mM MnCl_2_ (Sigma-Aldrich, 7773–01-5), 0.7 mM dNTPs with 10 U SuperScript II reverse transcriptase (Invitrogen, 18,064,022). The obtained cDNA products were purified using Mag-Bind Total Pure NGS beads (Omega Bio-Tek, M1378-01). Each sample was amplified separately by two PCRs using Q5 high-fidelity polymerase (NEB, M0149S) and zone-specific PCR primers. PCR 1 included 15 cycles: 98°C, 30 s; 98°C, 10 s; 55°C, 30 s; 72°C, 30 s; 72°C, 2 min. The obtained products were checked on 0.8% agarose gel, purified using Mag-Bind Total Pure NGS beads, and subjected to the second round of PCR amplification using 15 cycles: 98°C, 30 s; 98°C, 10 s; 55°C, 30 s; 72°C, 30 s; 72°C, 2 min. The final libraries were purified on 2% preparative agarose gel by Monarch DNA Gel Extraction kit (NEB, T1020L). The concentration of each library was measured by performing quantitative PCR (qPCR) using PerfeCTa® NGS Quantification Kit (Quanta Biosciences 10029–558) and following the manufacturer’s protocol. Purified amplicon libraries were pooled and sequenced on Illumina MiSeq Instrument.

### SHAPE-assisted structure predictions

The dataset obtained from Illumina MiSeq was saved into separate FASTQ files for each of the respective zones and reactions. A custom Python script ‘trimmer.py’ was used to trim raw sequences and remove sequencing artefacts. The FASTQ files corresponding to the (+), (-) and DC reactions were concatenated and processed by ShapeMapper v1.2 [66] to obtain mutation counts, sequence read depth and normalized reactivity values in graphical and CSV format, which help detect potential experimental issues. SuperFold v1.1 [29] was utilized to determine the partition function, minimum free energy and structural analysis. Shannon entropy was computed from base-pairing probabilities analysed during SuperFold partition function calculation with a partition Window size of 1200 nts and step size of 100 nts. The local median SHAPE reactivity and Shannon Entropy were calculated over a 55 nt sliding window with a fold step size of 300. RNAstructure v6.0 [67] was used for generating the secondary structure models by incorporating SHAPE reactivities as pseudo-energy constraints using slope and intercept values of 2.6 and −0.8, respectively. To determine the presence of a pseudoknot in the IRES RNA, we incorporated the reactivity data into the SHAPEknot v5.8.1 algorithm with the default parameters of SHAPEintercept = −0.8 and SHAPEslope = 2.6. VARNA applet v3-93, ViennaRNA’s RNAplot v2.4.17 and R-chie webserver [68] were used to visualize RNA secondary structures and arc plots.

### Comparative genomics screen within the genus *Pestivirus*

Complete genomes of all ICTV-listed *Pestivirus* species (A-K), as well as two unclassified Pestiviruses, i.e. Linda virus and Norway rat *Pestivirus* were downloaded from the NCBI GenBank database. The cmsearch tool from the Infernal suite v1.1.4 [69] was used to screen the Rfam covariance model RF00209 (*Pestivirus* internal ribosome entry site) against the library of 13 *Pestivirus* genomes, yielding structurally homologous elements within the 5′ UTR in each genome (Table S4). These were aligned with mafft v7.475 [70] and subjected to consensus structure prediction with RNAalifold v2.4.17 [71] from the ViennaRNA Package v2.4.17 [72]. As RNAalifold cannot directly predict structures that contain pseudoknots, a consensus structure was predicted in two steps. First, a nested, i.e. pseudoknot-free consensus structure was predicted, thereby constraining the regions involved in pseudoknot formation to being unpaired. In a second step, only the pseudoknot was allowed to form, with all other base pairs constrained to being unpaired. A combination of the two predictions yielded the anticipated consensus structure that involves an H-type pseudoknot. Statistical significance of covariation was assessed with R-scape v1.5.10 [73].

### Three-dimensional (3D) structure predictions of BVDV IRES RNA

RNA tertiary structure predictions were performed on a trimmed 106 nt construct that encompasses the portion of the BVDV1-NADL IRES that is involved in pseudoknot formation. The construct was verified to form the expected H-type pseudoknot with pKiss v2.2.13 [74]. To test whether the predicted H-type pseudoknot is sterically feasible in 3D, we used ernwin v1.0.1, a tool for sampling coarse-grained 3D structures from fragments of known RNA tertiary structures. Moreover, we performed 3D structure predictions with SimRNA v3.20. This Monte Carlo sampling approach uses a five-bead system per nucleotide to model a coarse-grained representation of RNA structure and employs an empirically derived knowledge-based potential. SimRNA was run for 16 million iterations in replica-exchange mode. The resulting trajectories were subjected to a clustering procedure, thereby filtering sets of similar structures in terms of root mean square deviation (RMSD) within 1% of all trajectories with the lowest energy. Two sets of low-energy candidate structures, comprising 17 and 21 structures, were then evaluated for specific helix angles with forgi v2.0 [54], a library for analysing RNA tertiary structures. The coarse-grained representations from ernwin and SimRNA, together with the structure probing data, allowed us to assess the feasibility and the structural characteristics of the detected pseudoknot. RNA 3D structures were visualized in PyMol v2.4.2 [75].

## Supplementary Material

Supplemental Material

## Data Availability

Custom R and Python scripts for data analysis are available at https://github.com/jzs0165/BVDV-Files

## Accession numbers

The high-throughput sequencing data generated in this study are deposited into SRA databases with bio-sample accession number: PRJNA725417, BVDV-NADL accession number: NC_011461.
