# Supplementary material for: Insights into the secondary and tertiary structure of the Bovine Viral Diarrhea Virus Internal Ribosome Entry Site
Source: RNA Biol. 2022 Apr 5;19(1):496–506. doi: 10.1080/15476286.2022.2058818 (PMC8986297; doi:10.1080/15476286.2022.2058818)
Supplement: Supplemental Material [file KRNB_A_2058818_SM1240.zip › supplementary/Supplementary Material_corrected.docx]

SUPPLEMENTAL MATERIAL

**Insights into the secondary and tertiary structure of the Bovine Viral Diarrhea Virus Internal Ribosome Entry Site**

Devadatta Gosavi^a^, Iwona Wower^b^, Irene K. Beckmann^c^, Ivo L. Hofacker^c,d^, Jacek Wower^b^, Michael T. Wolfinger^c,d*^, Joanna Sztuba-Solinska^a^*^*^*

*^a^ Department of Biological Sciences, Auburn University, 120 W. Samford Ave, Rouse Life Sciences Building, Auburn, AL 36849, United States; ^b^Department of Animal and Dairy Sciences, Auburn University, 209 Upchurch Hall, Auburn, AL 36849, United States; ^c^Department of Theoretical Chemistry, University of Vienna, Währingerstraße 17,1090 Vienna, Austria; ^d^Research Group Bioinformatics and Computational Biology, Faculty of Computer Science, University of Vienna, Währingerstraße 29,1090 Vienna, Austria.*

^*^To whom correspondence should be addressed J.S.S. Tel: +1-334-844-4830, Email: [jzs0165@auburn.edu](mailto:jzs0165@auburn.edu)

Correspondence may also be addressed to M.T.W. Tel: +43-1-4277-52747, Email: [michael.wolfinger@univie.ac.at](mailto:michael.wolfinger@univie.ac.at)

**Supplemental Tables**

**Table S1:** The structural context and nucleotide position of hyperreactive residues mapped within BVDV IRES RNA. The hyperreactive residues shared between short (S) and long (L) RNA constructs are indicated with asterisk.

| Domain | Structural Context | Nucleotide position | Reactivity values |
| --- | --- | --- | --- |
| I | SLIa, apical loop  SLIb, apical loop  SLIc, apical loop | A,26 (L)  G,22 (S)  A,54 (L)  G,58* (L,S)  A,60 (L)  G,63 (L)  G,58 (S)  A,91* (L,S)  A,99 (L) | 2.042881  2.421451  3.112186  2.754795, 2.007319  2.000723  2.083034  2.007319  2.205514, 2.156971  2.04383 |
|  | SS region between SL Ic and SL II | A,116 (L) | 2.742492 |
| III | SLIIIb, A-U paired region  SLIIIb, bulge  SS region between SL IIId1 and SL SLIIId2, three-way junction | A,271 (L)  A,272 (S)  A,273 (L)  G,272 (S)  A,335 (L) | 2.320081  2.678050  2.023822  2.67805  2.209314 |
|  | PK | G,386 (S) | 2.921128 |
|  | SS region, junction | A,409 (L)  U,417* (L,S)  A,438 (L)  A,444 (L)  U,453 (S)  A,455 (L)  A,461* (L,S)  U,465 (L)  A,469 (L)  A,474 (L) | 2.555229  2.391094, 3.93809  2.400219  2.826414  2.608208  2.942058  2.781298, 3.08476  2.427806  2.709064  2.250557 |

**Table S2:** The list of oligonucleotide sequences used for Cloning IRES from BVDV-NADL into pIW-IRES(eMS2hp)-EGFP expression vector.

| Primer | Sequence 5′- 3′ |
| --- | --- |
| IRES-NADL-forward | GTATACGAGAATTAGAAAAGGCACTCGTATACG |
| IRES-NADL-reverse | CATAAACAGGTTCCTCCACCCC |
| T7-IRES-for-3 | ATACGACTCACTATAGGGTACCGTATACGAGAATTAGAAAAGGCACTC |
| T7-NADL-reverse | GCCTGCAGTCGACTCGAGAGGATCCcataaacaggttcctccacccc |
| T7-NADL-R17-reverse | GCCTGCAGTCGACTCGAGAGGATCCgacatgggtgatcctcatgtccataaacaggttcctccacccc |
| A-5′ EGFP adaptor | GATCCGAATTCAAAACCACAACC |
| B-5′-EGFP adaptor | /5′Phos/GTGGTTTTGAATTCG |
| C-3′ EGFP adaptor | GTACAAGTAAGATATCAAAGGGCC |
| D-3′-EGFP adaptor | /5′Phos/TTTGATATCTTACTT |
| R17L-forward | GGAGGAACCTGTTTATGGGA |
| R17L-reverse | GCACCACCCCGGTGAACA |
| gblock-R17L | GGAGGAACCTGTTTATGGGATCCATCATCATCATCGACATGAGGATCACCCATGTCAACGATGATGATGAATTCAAAACCACAACCATGGTGAGCAAGGGCGAGGAGCTGTTCACCGGGGTGGTGC |

**Table S3:** The list of oligonucleotide sequences used for SHAPE-MaP probing. The underlined sequences represent the Illumina adapter sequence, followed by the randomized nucleotide sequence (NNNNN), while the color-coded portions in red and blue represent the gene-specific sequence. Illumina index sequences are in bold.

| Oligonucleotide | Sequence 5′ – 3′ | Position (nts) |
| --- | --- | --- |
| RT | CCATAAACAGGTTCCTCCACCCCGAC | 480-505 |
| Z1 Forward | GACTGGAGTTCAGACGTGTGCTCTTCCGATCTNNNNNGGGAGACCCAAGCTGGC | 1-17 |
| Z1 Reverse | CCCTACACGACGCTCTTCCGATCTNNNNNCCACGTGGCATCTCGAGACC | 264-283 |
| Z2 Forward | GACTGGAGTTCAGACGTGTGCTCTTCCGATCTNNNNNGCCCTGAGTACAGGGTAGTC | 217-236 |
| Z2 Reverse | CCCTACACGACGCTCTTCCGATCTNNNNNCATAAACAGGTTCCTCCACC | 484-504 |
| Illumina P5 index adapter | AATGATACGGCGACCACCGAGATCTACACTCTTTCCCTACACGACGCTCTTCCG | N/A |
| Illumina P7 index adapter (I-1) | CAAGCAGAAGACGGCATACGAGAT**CGTGAT**GTGACTGGAGTTCAGAC | N/A |
| Illumina P7 index adapter (I-2) | CAAGCAGAAGACGGCATACGAGAT**ACATCG**GTGACTGGAGTTCAGAC | N/A |
| Illumina P7 index adapter (I-3) | CAAGCAGAAGACGGCATACGAGAT**GCCTAA**GTGACTGGAGTTCAGAC | N/A |
| Illumina P7 index adapter (I-4) | CAAGCAGAAGACGGCATACGAGAT**TGGTCA**GTGACTGGAGTTCAGAC | N/A |
| Illumina P7 index adapter (I-5) | CAAGCAGAAGACGGCATACGAGAT**CACTGT**GTGACTGGAGTTCAGAC | N/A |
| Illumina P7 index adapter (I-6) | CAAGCAGAAGACGGCATACGAGAT**ATTGGC**GTGACTGGAGTTCAGAC | N/A |
| Illumina P7 index adapter (I-7) | CAAGCAGAAGACGGCATACGAGAT**GATCTG**GTGACTGGAGTTCAGAC | N/A |
| Illumina P7 index adapter (I-8) | CAAGCAGAAGACGGCATACGAGAT**TCAAGT**GTGACTGGAGTTCAGAC | N/A |
| Illumina P7 index adapter (I-9) | CAAGCAGAAGACGGCATACGAGAT**CTGATC**GTGACTGGAGTTCAGAC | N/A |
| Illumina P7 index adapter (I-10) | CAAGCAGAAGACGGCATACGAGAT**AAGCTA**GTGACTGGAGTTCAGAC | N/A |
| Illumina P7 index adapter (I-11) | CAAGCAGAAGACGGCATACGAGAT**GTAGCCC**GTGACTGGAGTTCAGAC | N/A |
| Illumina P7 index adapter (I-12) | CAAGCAGAAGACGGCATACGAGAT**TACAAG**GTGACTGGAGTTCAGAC | N/A |

**Table S4:** Genomic coordinates of the IRES Domain III region in representative isolates of Pestivirus species A-K and two unassigned Pestiviruses.

| Virus | Accession | Coordinates |
| --- | --- | --- |
| Pestivirus A | NC_001461.1 | 116-393 |
| Pestivirus B | NC_039237.1 | 116-393 |
| Pestivirus C | NC_002657.1 | 108-381 |
| Pestivirus D | NC_003679.1 | 106-380 |
| Pestivirus E | NC_024018.2 | 107-377 |
| Pestivirus F | NC_003678.1 | 124-406 |
| Pestivirus G | NC_003678.1 | 110-390 |
| Pestivirus H | NC_012812.1 | 113-391 |
| Pestivirus I | NC_018713.1 | 107-385 |
| Pestivirus J | KJ950914.1 | 122-406 |
| Pestivirus K | KY652092.1 | 94-386 |
| Linda Virus | NC_035432.1 | 107-389 |
| Norway rat pestivirus | NC_025677.1 | 122-406 |


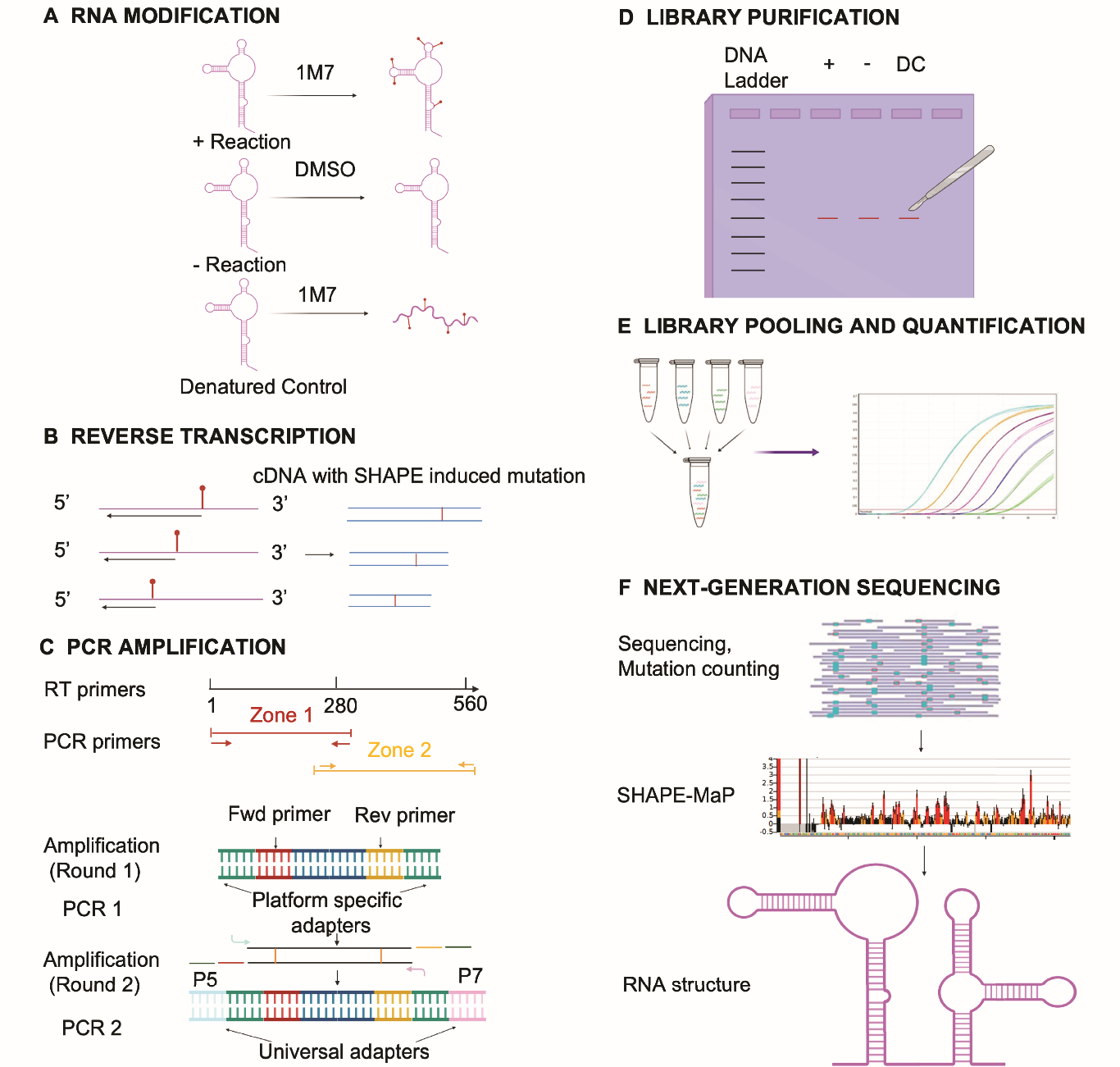
**Supplemental Figures**

**Figure S1.** Overview of the SHAPE-MaP probing technique. (**A**) The probing experiments include three reactions: positive (+), in which RNA is modified with electrophilic reagent; negative (-), in which RNA is treated with DMSO (vehicle); and denaturing control, which includes denatured and modified RNA. (**B**) Reverse transcription of modified RNA results in cDNA products with mutational signatures that correspond to the nucleotide reactivities. (**C**) Stepwise amplification of cDNA products results in the addition of custom adapters. (**D**) The PCR products are purified, (**E**) quantified, and pooled at a specific concentration. (**F**) The next-generation sequencing results in mutation counts that reflect reactivity values for each residue. These values are used as pseudo-energy constraints to guide the secondary structure prediction of target RNA.


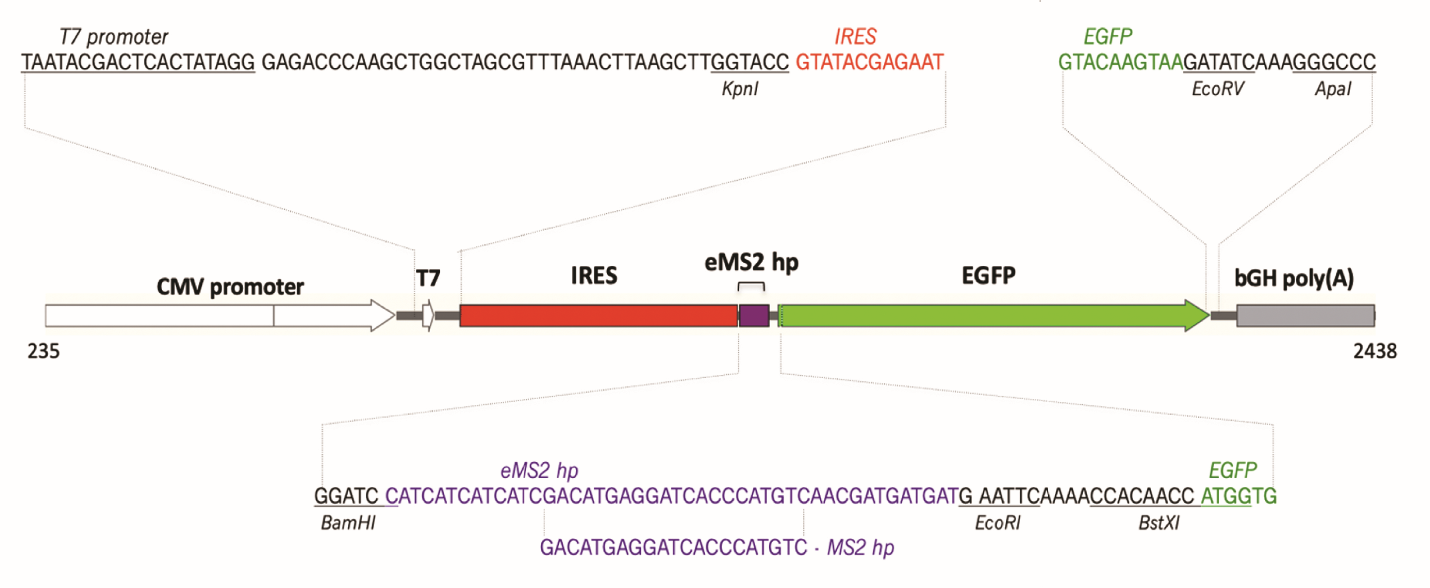


**Figure S2.** Construction of plasmid pIW-IRES(eMS2hp)-EGFP. The IRES and EGFP encoding genes are under the control of CMV promoter. Restriction EcoRI and BstXI sites are indicated and used to generate templates for *in vitro* transcription of IRES RNA tagged with eMS2 hairpin and a longer mRNA encoding IRES RNA, MS2 hairpin and EGFP.


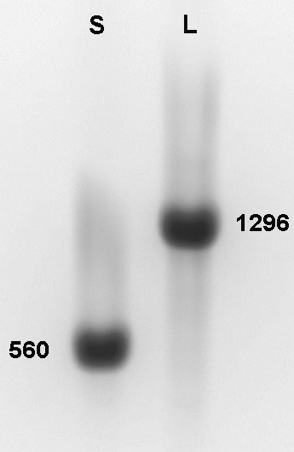


**Figure S3.** Native agarose gel electrophoresis of BVDV IRES short (S) (lane 1) and long (L) RNA constructs (lane 2). Sizes of each transcript are indicated (S – 560, L – 1296 nts).


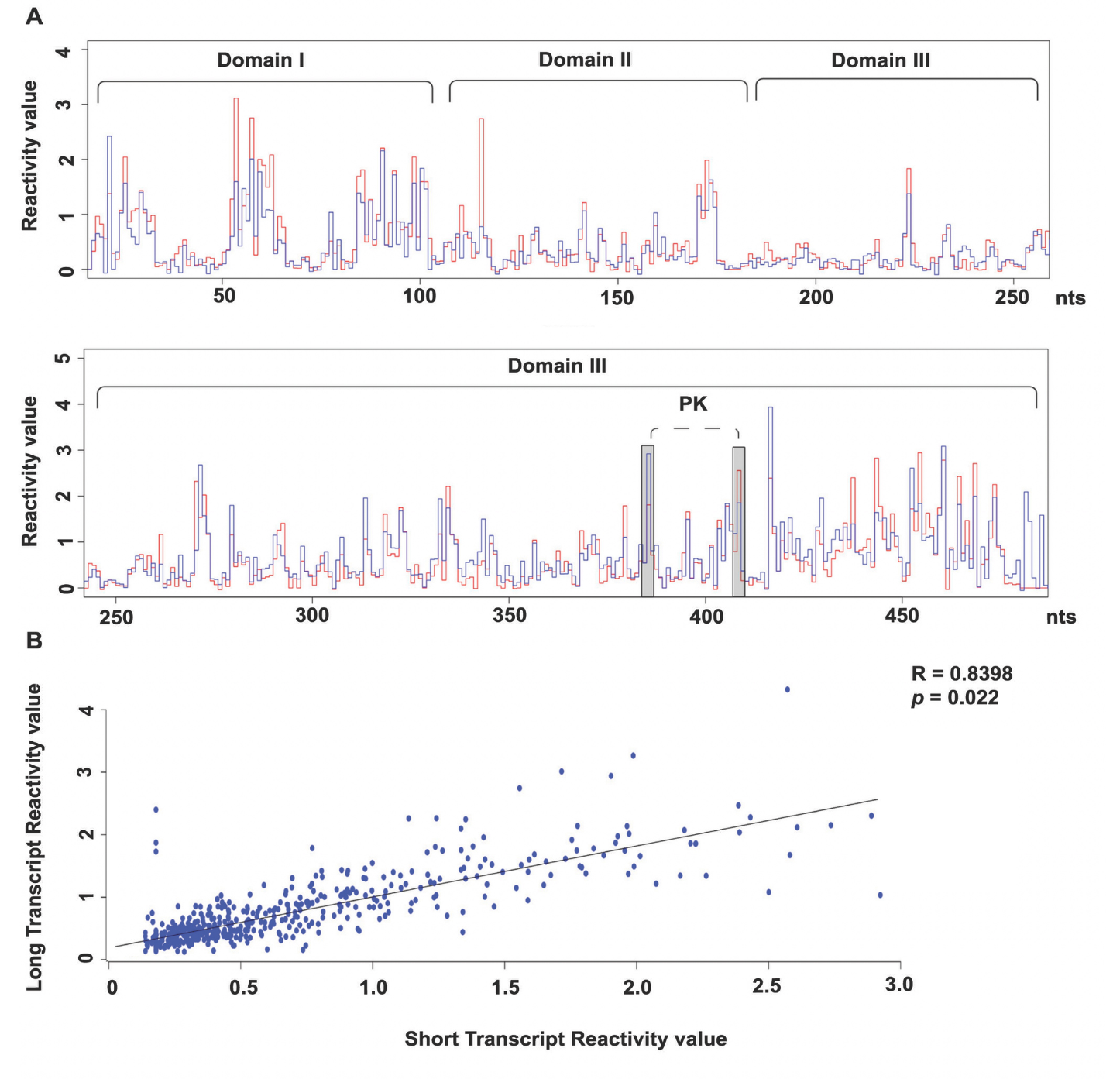


**Figure S4.** (**A**) Step plots representing the quantitative comparison of SHAPE-MaP reactivity profiles obtained for the short (red) and long (blue) BVDV IRES RNA constructs. The X-axis represents the nucleotide positions. The Y-axis corresponds to the SHAPE-MaP reactivity values. The plots are divided into two zones: 1 (nts 1-283) and 2 (nts 217-504). The boxed regions highlight the single-stranded regions involved in the formation of a pseudoknot (PK). (**B**) Scatter graph representing a positive correlation between the SHAPE-MaP reactivity values obtained for the BVDV IRES long transcript (Y-axis) against the reactivity values of BVDV IRES short transcript (X-axis). Pearson’s correlation coefficient R = 0.8398 and probability, p-value=0.022, are indicated on the graph.


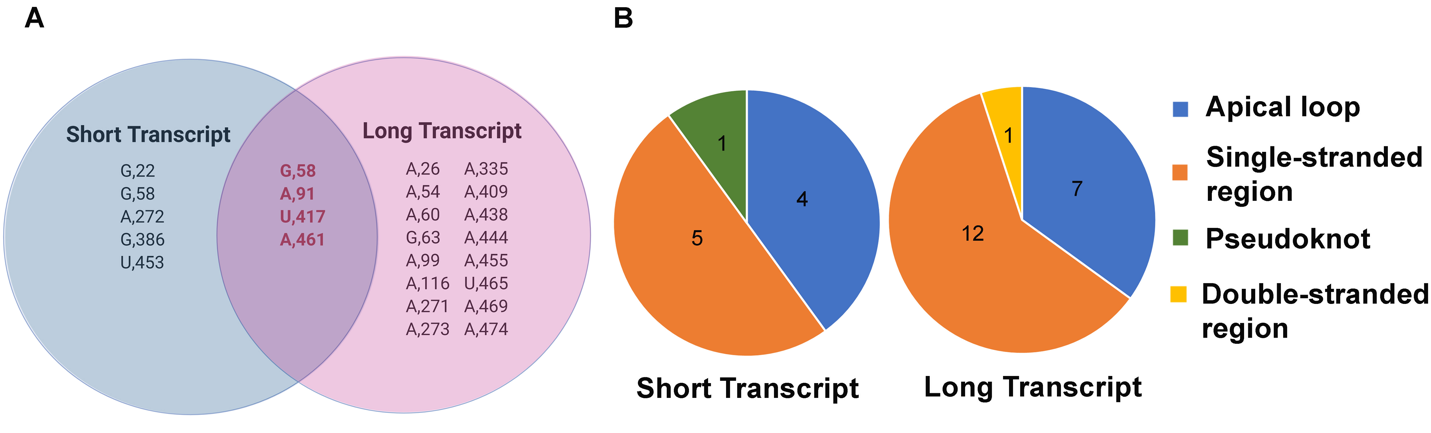


**Figure S5.** (**A**) Venn diagram indicating hyperreactive nucleotides and their positions in short and long BVDV IRES RNAs. (**B**) Pie charts showing the structural context of hyperactive residues.


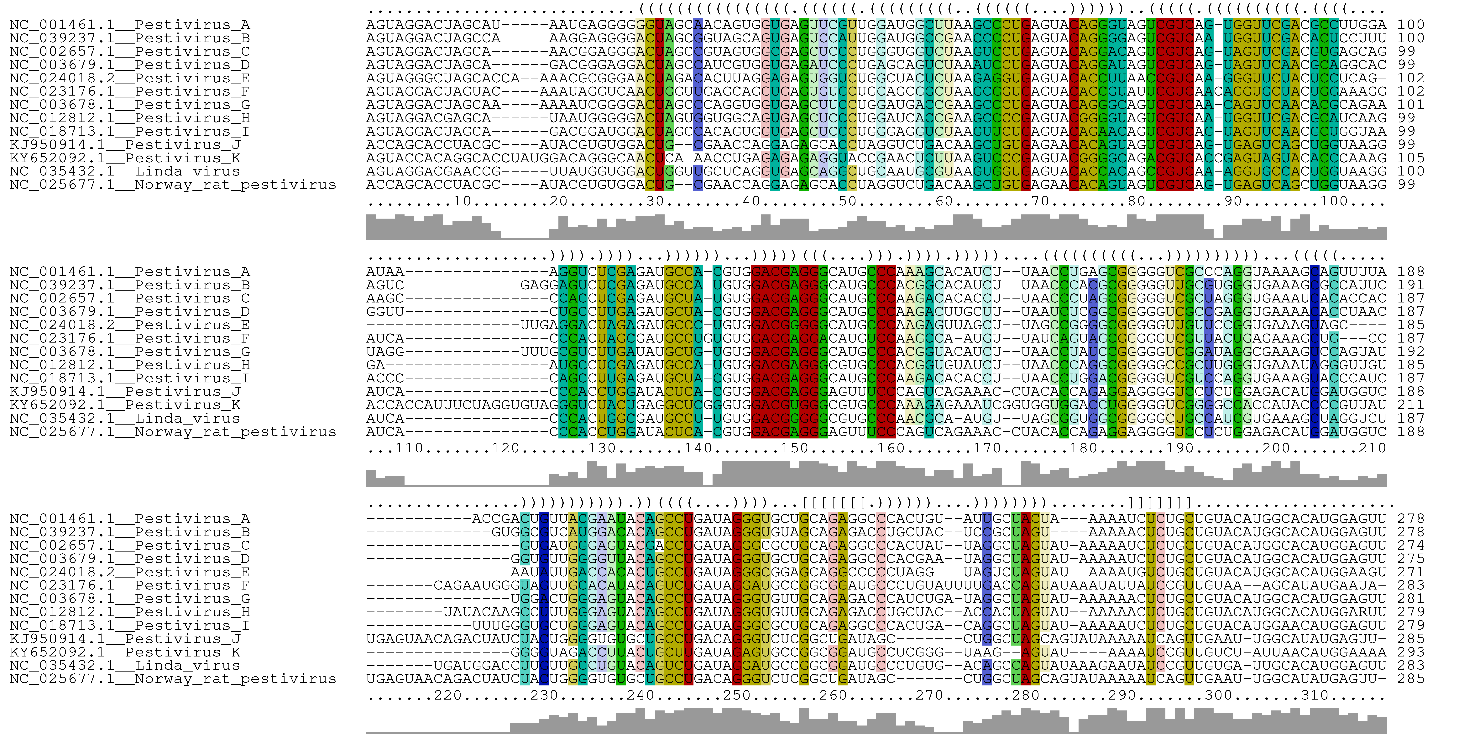


**Figure S6.** Multiple sequence alignment of the IRES Domain III generated for 13 Pestiviruses that has been used to compute the consensus structure shown in Figure 3. Color coding of individual columns and base pairs follows the RNAalifold color scheme (insert in Figure 3). Grey bars below the alignment highlight sequence conservation levels. The consensus structure is plotted above the alignment in dot-bracket notation. The pseudoknot is shown in the bottom block with square brackets.


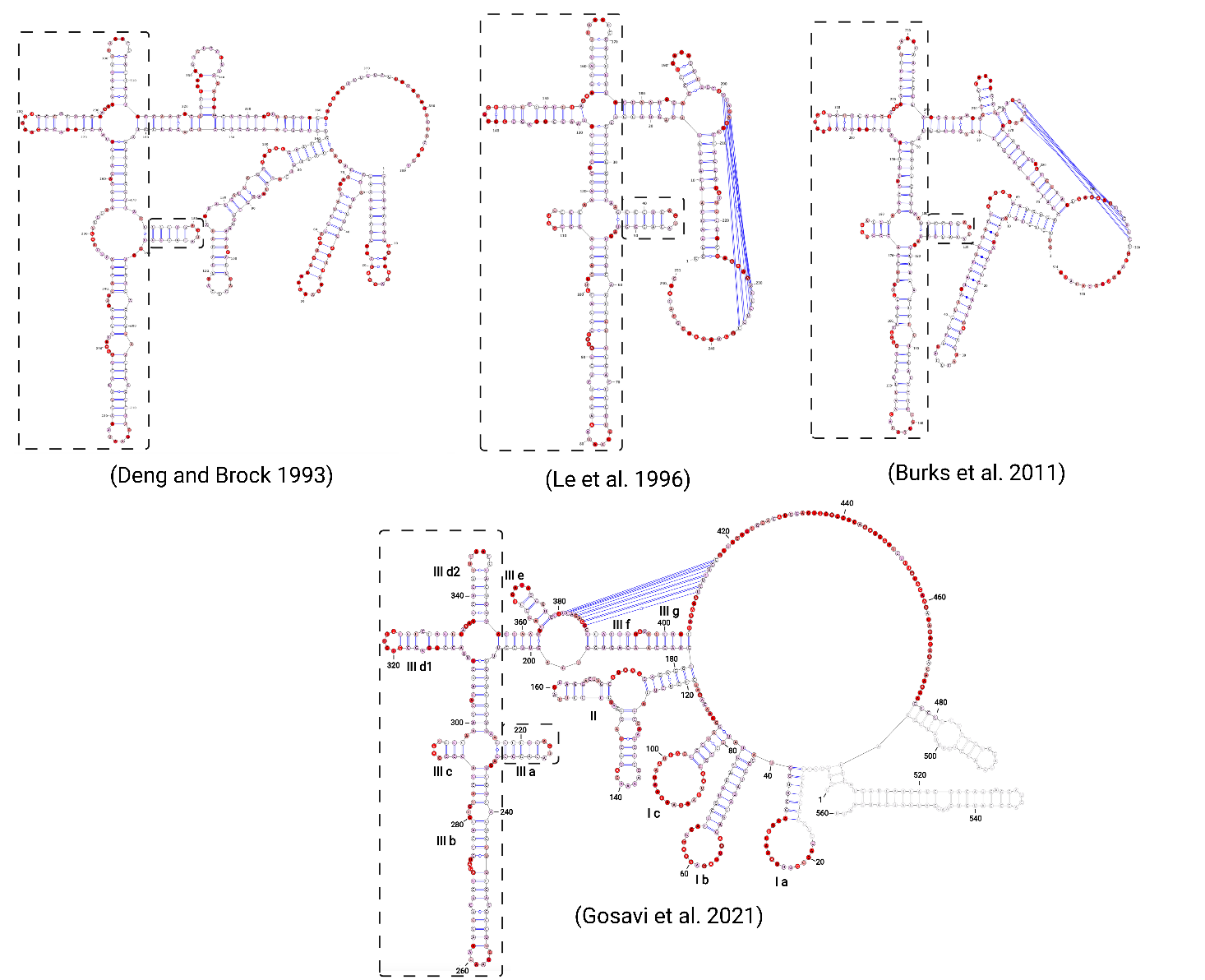


**Figure S7.** Comparative structural depiction of our SHAPE-assisted BVDV IRES RNA structure model and previously proposed models indicating high structural similarity of Domain III as indicated by dashed-box. SHAPE reactivity data obtained from our experiments has been overlaid on all structure models.


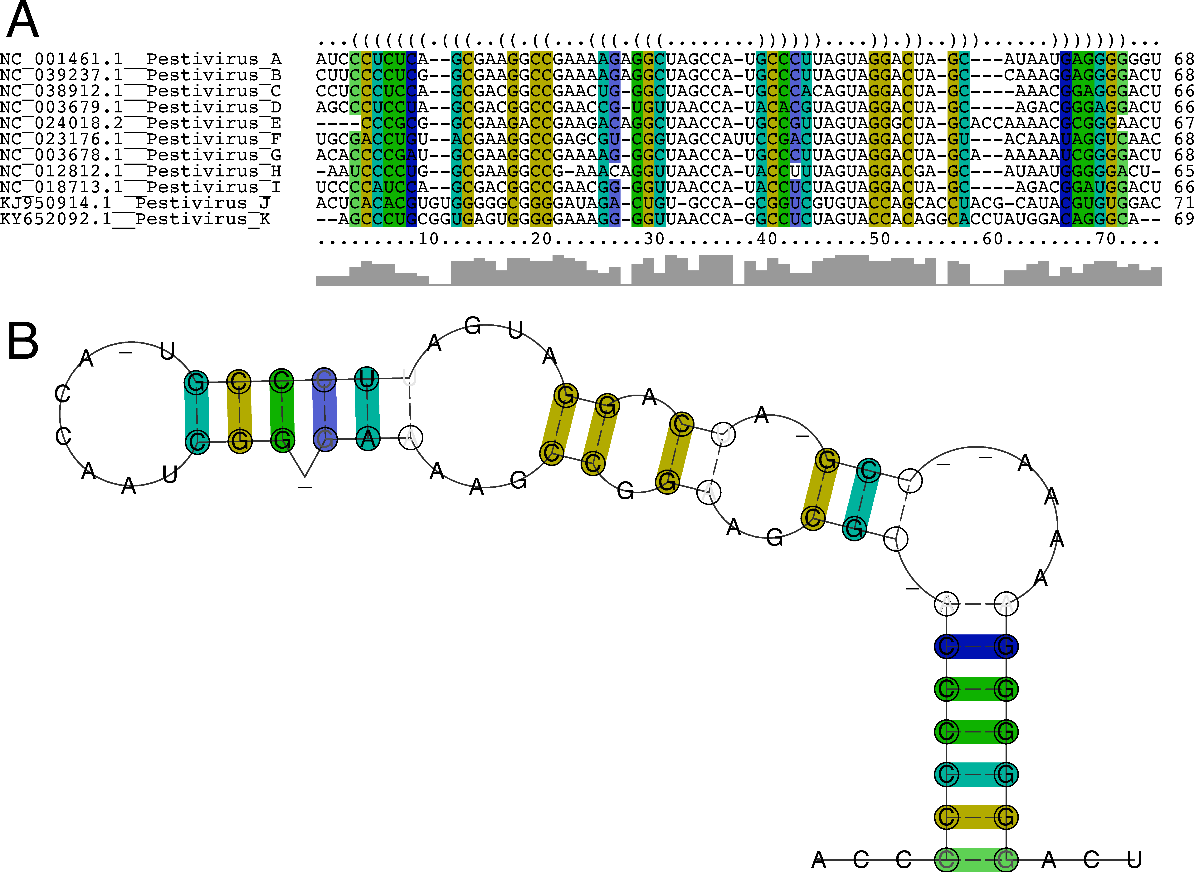


**Figure S8.** Consensus structure prediction of Pestivirus IRES Domain II, shown as alignment (**A**) and consensus minimum free energy structure (**B**), computed by RNAalifold. The alignment has been built from homologous sequences from Pestivirus species A-K. BVDV NADL is represented by the first sequence in the alignment, NC_001461.1__Pestivirus_A. Coloring of paired columns in the alignment matches with stacked regions in the consensus structure, and indicates different levels of covariation. See also Figure 3 for an explanation of the color scheme. A grey bar below the alignment in (**A**) indicates the level of primary sequence conservation.


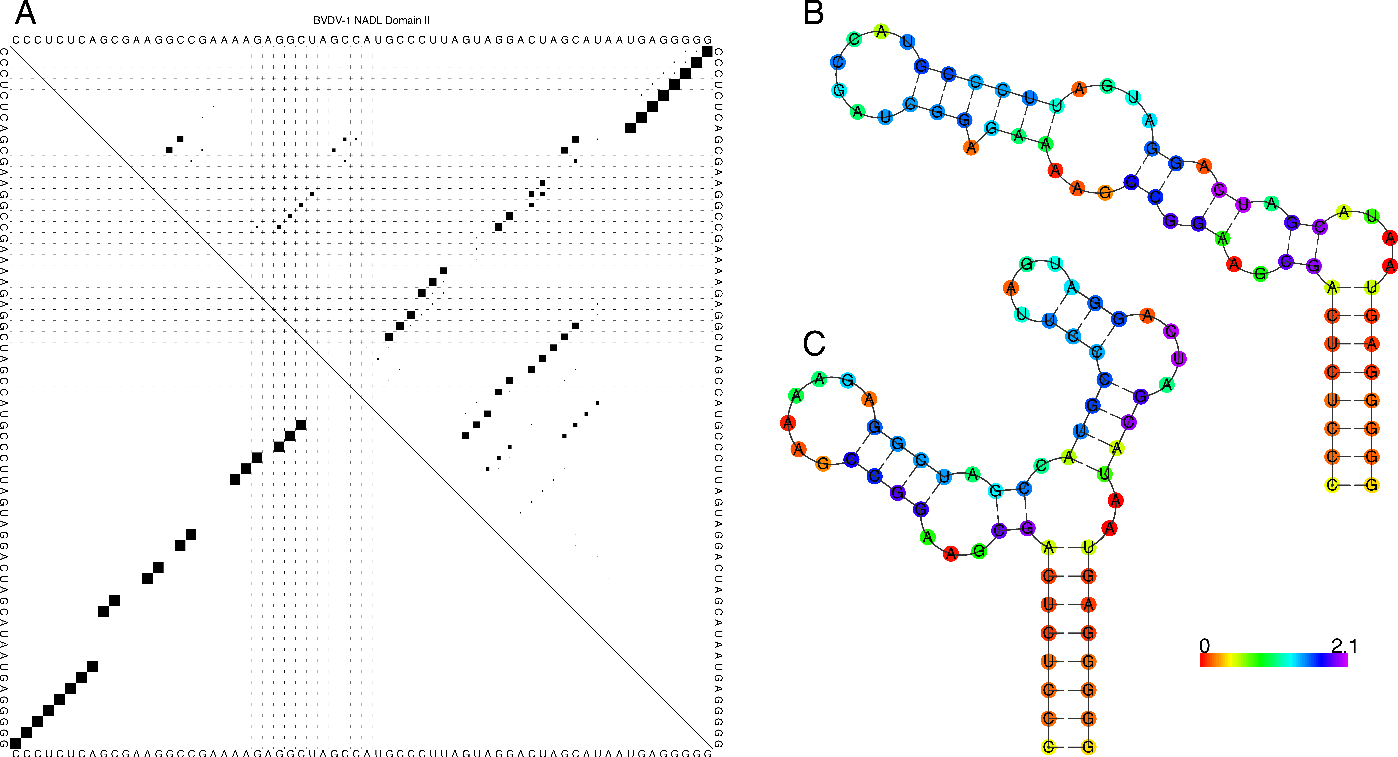


**Figure S9.** (**A**) Base pairing probability (BPP) dot plot of BVDV NADL domain II. BPP of a particular interaction is proportional to the area of the dots, where the upper triangle represents all interactions that are possible in the thermodynamic ensemble, whereas the lower triangle depicts only pairing probabilities of the minimum free energy structure (MFE).Substantial BPP in the structural ensemble, that is not observed in the MFE structure highlights large conformational flexibility of this element. (**B**) Minimum free energy structure (energy: -22.0 kcal/mol). (**C**) Alternative Y-shaped structure (energy: -21.0 kcal/mol). Overlay colors in (**B**) and (**C**) show positional entropy values (Gruber et al. 2008), indicating high structural flexibility of the central part of BVDV IRES domain II.


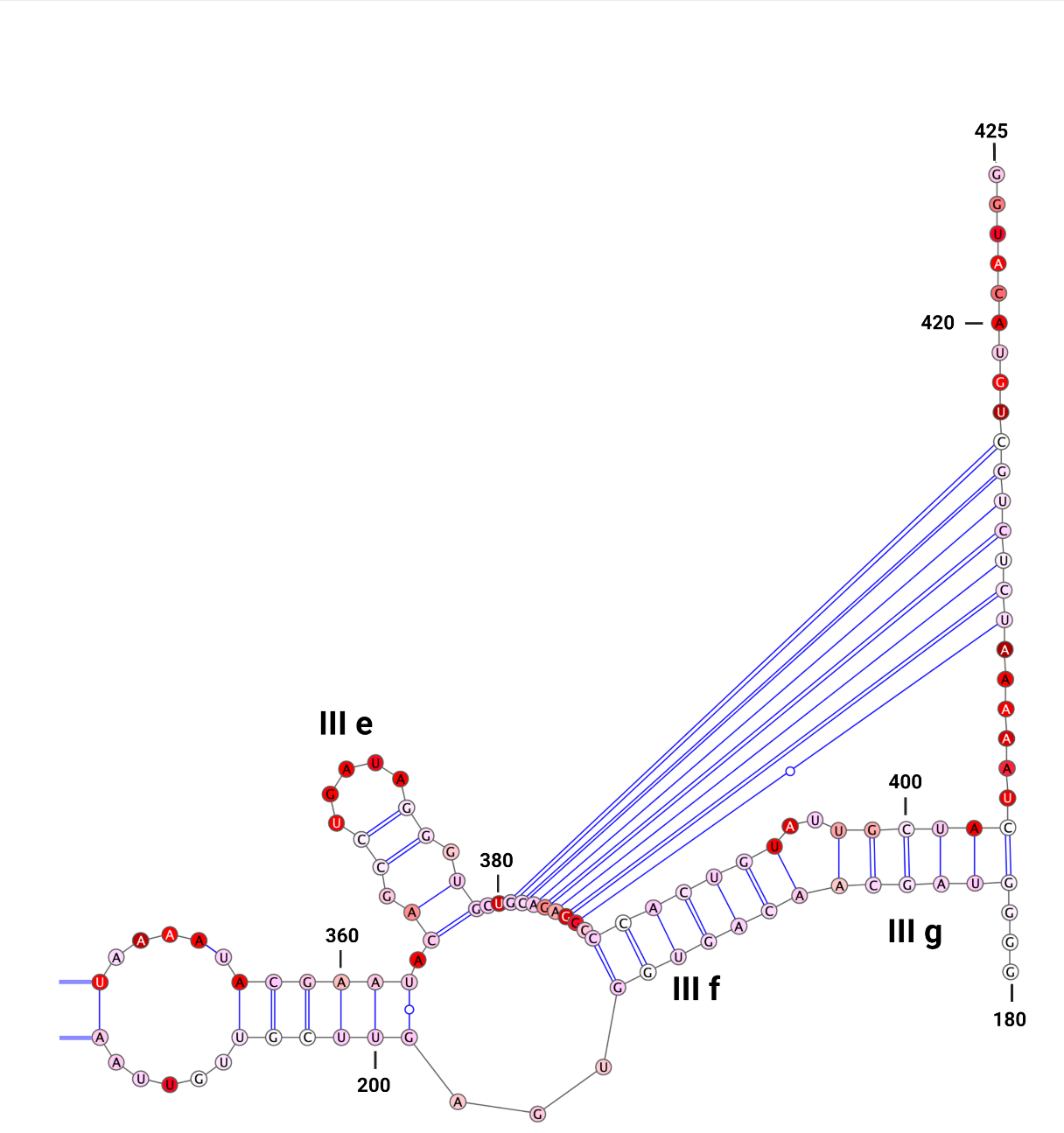


**Figure S10.** BVDVsegment_180_425 illustrating the existence of an H-type pseudoknot near the basal stem of Domain III.
